# Complete mitochondrial genomes from two species of Chinese freshwater crabs of the genus *Sinopotamon* recovered using next-generation sequencing reveal a novel gene order (Brachyura, Potamidae)

**DOI:** 10.3897/zookeys.705.11852

**Published:** 2017-10-02

**Authors:** Xing Yuhui, Zhou Lijun, Hou Yue, Wang Xiaoqi, Zhang Chen, Zhang Huilun, Wang Ruoran, Pan Da, Sun Hongying

**Affiliations:** 1 Jiangsu Key Laboratory for Biodiversity and Biotechnology,; 2 College of Life Sciences, Nanjing Normal University, Nanjing 210023, China; 3 College of Life Sciences, Nanjing Normal University, Nanjing 210023, China

**Keywords:** Eubrachyuran, gene rearrangement, mitochondrial genome, primary freshwater crab, *Sinopotamon*

## Abstract

Recent morphological and molecular evidence has challenged classical interpretations of eubrachyuran phylogeny and evolution. Complete mitochondrial genomes of two species of potamid freshwater crabs, *Sinopotamon yaanense* and *Sinopotamon yangtsekiense* were obtained using next-generation sequencing. The results revealed a novel gene order with translocations of a five-gene block and a tRNA gene in comparison to available brachyuran mitochondrial genomes. DNA sequence comparisons position the Potamidae, a primary freshwater crab family, outside of the clade for the traditional heterotreme families, and closer to the clade that includes the thoracotreme families of grapsoid and ocypodoid crabs. Mitogenomic comparisons using rapid next-generation sequencing and a much wider taxonomic sample are required for a high-resolution examination of the phylogenetic relationships within the Eubrachyura.

## Introduction

Brachyuran crabs are one of the most species-rich and economically important groups in extant crustaceans with about 7200 species described (Castro et al. 2015), of which two major groups, Heterotremata and Thoracotremata, are collectively referred to as the Eubrachyura (Guinot 1977; de Saint-Laurent 1980; Ahyong et al. 2007; Tsang et al. 2014). However, interrelationships of eubrachyurans still remain controversial when considering published nuclear DNA and/or mitochondrial DNA (mtDNA) and morphological analyses, especially regarding relationships among families of primary freshwater crabs and between them and other eubrachyurans (Schubart et al. 2000; Von Sternberg and Cumberlidge 2001; Cumberlidge et al. 2008; Ji et al. 2014; Tsang et al. 2014; Xing et al. 2016). Inadequate taxon sampling and phylogenetic data may mislead tree reconstruction. Mitochondrial genome (mitogenome) contains rich signals from both sequence and arrangement of 13 protein-coding genes (PCG), two rRNA genes, 22 tRNA genes and an AT-rich region (main non-coding region, mNCR) in a closed circular DNA molecule (Boore et al. 1998; Boore 1999; Sun et al. 2003). Therefore, they are considered powerful markers for resolving ancient phylogenetic relationships (Boore 1999). Nevertheless, the number of complete mitogenomes of brachyurans is limited to 52 species published to date, which is not commensurate with the extant species diversity for the Brachyura (including several species with incomplete mitogenomes; Yamauchi et al. 2003; Segawa and Aotsuka 2005; Sun et al. 2005; Ji et al. 2014; Shi et al. 2015; Wang et al. 2016; Xing et al. 2016). Meanwhile, knowledge of mitogenomes from primary freshwater crabs remains scant, and only three species have been sampled (including an incomplete sequence; Segawa and Aotsuka 2005; Ji et al. 2014; Wang et al. 2016).

Next-generation sequencing (NGS), combined with bioinformatic annotation, is becoming increasingly common for recovering animal mitogenome sequences and allows a rapid amplification-free sequencing (Jex et al. 2010). It has been used for recovering entire mitogenomes of decapod crustaceans, including brachyurans (Gan et al. 2014a, b, c, d, e; Tan et al. 2015; Xing et al. 2016). In the present study, two new mitogenomes of *Sinopotamon* species, *S. yaanense* (Chung and Ts’ao, 1962) and *S. yangtsekiense* Bott, 1967 from the family Potamidae are reported using NGS through two different strategies, transcriptome and total genomic DNA, respectively. The gene arrangements for the two mitogenomes were then compared with those of available brachyuran mitogenomes.

*Sinopotamon yaanense* and *S. yangtsekiense*, as two representatives of the endemic genus *Sinopotamon* occur in China, are distributed in the middle and lower reaches of the Yangtze River Basin, respectively (Dai 1999; Fang et al. 2013). When refer to the phylogenetic relationships among *Sinopotamon* species (Ji et al. 2016), the former belongs to a distinct clade ranged in Sichuan Basin and its surrounding mountains, with rounded lobes of the last segment of the male first gonopods (G1) (Dai 1999; Ji et al. 2016). While the latter species have acute lobes of the last segment of G1, is clustered together with those occurring in middle reaches of the Yellow River and the upper and middle reaches of the Huaihe River (Fang et al. 2013, 2015; Ji et al. 2016). Along with the relationships reconstructed by morphological data and DNA barcoding involving broader taxon sampling (Zhou et al. unpublished), the monophyly of *Sinopotamon* crabs is therefore challenged (Ji et al. 2016; Shih et al. 2016). In this study, we provide a thorough description of mitogenomes of these two *Sinopotamon* species, and present independent molecular evidence for phylogenetic relationships among related groups.

## Materials and methods

### Sample collection

Specimens of *S. yaanense* and *S. yangtsekiense* were collected by hand from mountain streams of the Emei Mountain in Sichuan Province, China (29°36'3﻿﻿﻿﻿"N; 103°19'59"E) and Ningguo, Anhui Province, China (30°38'21"N; 118°59'27"E), respectively. These specimens were preserved in 95% ethanol and identified utilizing morphological information presented in Dai (1999) under a stereo dissection microscope (Nikon SMZ645). Voucher specimens of *S. yaanense* (catalogue number EMMZ06) and *S. yangtsekiense* (catalogue number CJ01) were deposited in the Jiangsu Key Laboratory for Biodiversity and Biotechnology, College of Life Sciences, Nanjing Normal University (NNU), Nanjing, China.

### RNA and DNA extraction, genome sequencing, and PCR

Next-generation transcriptome sequencing and next-generation total genomic DNA sequencing were used to obtain mitogenomes of the two *Sinopotamon* species. For transcriptome sequencing, total RNA of *S. yangtsekiense* was extracted from fresh tissue of one individual using the TRIzol (Takara). After determining the RNA quality, the sample was enriched by Oligo (dT) and broken into short RNA fragments. The cDNA library was then prepared and sequenced on the Illumina Hi-Seq 2000 platform (BGI). All raw data were processed to remove adaptors and clean data were assembled de novo using Trinity (Grabherr et al. 2011). Finally, unigene sequences resulting from the assemblies were identified by BLAST alignment against the nucleotide database at the National Center for Biotechnology Information (NCBI) or by comparison with other published brachyuran sequences.

For total genomic DNA sequencing, total genomic DNA of *S. yaanense* was extracted using Cell and Tissue DNA Extraction Kit (Generay Biotech). The samples were sequenced on the Illumina HiSeq 4000 platform (BGI). The sequencing libraries with average insert sizes of approximately 300 bp were prepared, and then sequenced as 150 bp paired-end runs (about 2 Gb raw data each species). De novo assemblies were conducted with Geneious 9.1.4 using the Map to Reference program (Kearse et al. 2012) with parameter settings (Minimum Overlap = 30~50, Minimum Overlap Identity: 80~100, Maximum Mismatches Per Read = 10%). The mitochondrial contigs were then extracted and identified using BLAST alignment against the closest reference mitogenome at NCBI. Sanger sequencing (Sanger and Coulson 1975) was also used to verify the gene fragments and to obtain fragments between two long contigs where some reads were not assembled.

### Mitogenome annotation and analyses

The assembled and identified mitochondrial DNA sequences were further annotated and analyzed. The locations of PCGs and rRNA genes were preliminarily annotated by DOGMA website (Wyman et al. 2004). The coding regions of PCGs were identified by using the NCBI ORF Finder (https://www.ncbi.nlm.nih.gov/orffinder/), and subsequently annotated by alignments of homologous genes of other published brachyuran mitogenomes. Codon usage of PCGs was determined by MEGA 6 (Tamura et al. 2013). Transfer RNA genes were mainly identified by tRNAscan-SE (Lowe and Eddy 1997); the remains were identified according to their tRNA-like secondary structures and anticodon sequences. Two rRNA genes were identified by alignment with other published brachyuran sequences. The nucleotide sequences of the complete mitogenomes for *S. yaanense* and *S. yangtsekiense* were deposited in the NCBI database under the Accession No. KY785880 and KY785879.

### Phylogenetic analysis

In-group and out-group taxa are listed in Table 1. After removing all termination codons, the putative amino acid (AA) sequences for each of the 13 mt PCGs and sequences for two rRNAs were individually aligned using MAFFT 7.215 (Katoh and Standley 2013) with the iterative refinement method G-INS-I (accurate alignment), in which the gap opening and extension penalties were 1.53 and 0.123, respectively. Ambiguous or randomly similar sites were removed by Aliscore 2.0 (Misof and Misof 2009; Kuck et al. 2010) using default settings. These AA alignments were later used as a backbone to align the corresponding nucleotide (NT) sequences using DAMBE 5.3.15 (Xia and Xie 2001). All 13 PCGs alignments were combined to create dataset A, and two rRNAs and 13 PCGs were concatenated to create dataset B. Subsequently, PartitionFinder 1.1.1 (Lanfear et al. 2012) was used to find the best-fit partitioning schemes and models using a greedy search with RAxML (Stamatakis 2006a) and Bayesian information criterion (BIC). Maximum likelihood (ML) analyses for all datasets were performed using RAxML-HPC2 on XSEDE 8.0.0 (Stamatakis 2014) through the Cipres Science Gateway (Miller et al. 2010). Given the advantages of Gamma over CAT when the sequences were less than 50 (Stamatakis 2006b) and the best-fit models calculated by the PartitionFinder 1.1.1 was not recommended by the developer of RAxML (Mayrose et al. 2005; Stamatakis 2006a), the model GTR + Gamma for all NT datasets were used. MrBayes 3.2.2 (Huelsenbeck and Ronquist 2001) was used to perform Bayesian inference (BI) analysis.

**Table 1.** Taxon sampled and its mitogenomic information used in this study.

	**Taxon**	**Species**	**Length (bp)**	**GenBank Accession No.**	**Reference**
**Ingroup**					
**Brachyura**	**Podotremata**				
	**Homoloidea**	*Homologenus malayensis*	15793	KJ612407	Unpublished
		*Moloha majora*	15903	KT182069	Shi et al. 2016
	**Raninoidea**				
		*Umalia orientalis*	15466	NC_026688	Shi et al. 2015
		*Ranina ranina*	15557	NC_023474	unpublished
		*Lyreidus brevifrons*	16112	NC_026721	Shi et al. 2015
	**Eubrachyura**				
	**Heterotremata**				
	**Portunoidea**				
		*Thalamita crenata*	15787	NC_024438	Tan et al. 2016a
		*Callinectes sapidus*	16263	NC_006281	Place et al. 2005
		*Portunus trituberculatus*	16026	NC_005037	Yamauchi et al. 2003
		*Charybdis japonica*	15738	NC_013246	Liu et al. 2010
		*Chaceon granulatus*	16135	NC_023476	unpublished
		*Scylla olivacea*	15723	NC_012569	unpublished
	**Majoidea**				
		*Chionoecetes japonicus*	15341	AB_735678	unpublished
	**Calappoidea**				
		*Ashtoret lunaris*	15807	NC_024435	Tan et al. 2016b
	**Eriphioidea**				
		*Myomenippe fornasinii*	15658	NC_024437	Tan et al. 2016c
	**Xanthoidea**				
		*Pseudocarcinus gigas*	15515	NC_006891	Miller et al. 2005
	**Bythograeoidea**				
		*Gandalfus yunohana*	15567	NC_013713	Yang et al. 2010
		*Austinograea alayseae*	15620	NC_020314	Yang et al. 2013
	**Potamoidea**				
		*Geothelphusa dehaani*	18197	NC_007379	Segawa et al. 2005
		*Sinopotamon xiushuiense*	18460	NC_029226	Wang et al. 2016
		*S. yangtsekiense*	17885	KY785879	Present study
		*S. yaanense*	17126	KY785880	Present study
	**Thoracotremata**				
	**Ocypodoidea**				
		*Ilyoplax deschampsi*	15460	NC_020040	Ji et al. 2014
		*Ocypode ceratophthalmus*	15564	NC_025324	Tan et al. 2016e
		*Mictyris longicarpus*	15548	LN_611670	Tan et al. 2016d
	**Grapsoidea**				
		*Eriocheir japonica sinensis*	16354	NC_006992	Sun et al. 2005
		*Xenograpsus testudinatus*	15798	NC_013480	Ki et al. 2009
		*Pachygrapsus crassipes*	15652	NC_021754	Yu et al. 2014
		*Cyclograpsus granulosus*	16300	NC_025571	Tan et al. 2016f
		*Chiromantes neglectum*	15920	KX156954	Xing et al. 2016
		*Parasesarma tripectinis*	15612	KU343209	Unpublished
		*Sesarmops sinensis*	15905	KR336554	Unpublished
		*Metopaulias depressus*	15765	KX118277	Unpublished
**Outgroup**					
**Anomura**	**Paguroidea**				
		*Pagurus longicarpus*	15630	NC_003058	Hickerson et al. 2000
		*Paralithodes brevipes*	16303	NC_021458	Unpublished
		*Lithodes nintokuae*	15731	NC_024202	Unpublished
	**Galatheoidea**	*Neopetrolisthes maculatus*	15324	NC_020024	Shen et al. 2013
		*Shinkaia crosnieri*	15182	NC_011013	Yang et al. 2008
**Thalassinidea**	**Gebiidea**				
		*Austinogebia eduli*s	15761	NC_019606	Lin et al. 2012
		*Upogebia major*	16119	JF793665	Kim et al. 2011a
**Polychelidae**	**Eryonoidea**				
		*Polycheles typhlop*s	16221	NC_020026	Shen et al. 2013
**Astacidea**	**Astacoidea**				
		*Cambaroides similis*	16220	NC_016925	Kim et al. 2012
	**Nephropoidea**				
		*Homarus americanus*	16432	NC_015607	Kim et al. 2011b
	**Parastacoidea**				
		*Cherax destructor*	15894	AY383557	Miller et al. 2004

## Results

### Mitogenome size and organization

**Figure 1.**
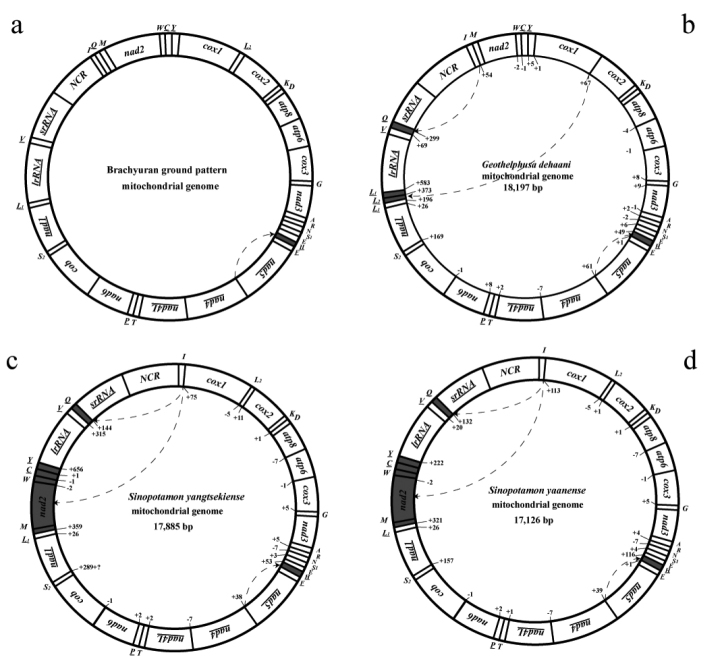
Mitochondrial genome sequenced in the present study. Gene order and sizes are shown relative to one another, including non-coding regions. Protein-coding genes encoded on the light strand are underlined. Transfer RNA (tRNA) genes encoded on the light strand are underlined. Each tRNA gene is designated by a single-letter amino acid code, except *L1* (*trnLeu (CUN)*), *L2* (*trnLeu (UUR)*), *S1* (*trnSer (AGN)*) and *S2* (*trnSer (UCN)*). Numbers inside circles represent the size of the non-coding region separating two adjacent genes or the amount of shared nucleotides between two overlapping genes. The translocations of gene or gene block are shaded gray.

The complete mitogenome sequences of *S. yaanense* and *S. yangtsekiense* were, respectively, 17,126 bp and 17,885 bp in length, between 15,612 bp and 18,460 bp typical in length for eubrachyurans (Table 1). Both sequences contained the entire set of 37 genes plus a mNCR (Fig. 1). The larger mitogenome size in *S. yangtsekiense* was due to greatly expanded non-coding nucleotides of intergenic spacers with 1,964 bp in total (ranging from 1 to 656 bp), but only 1,135 bp in *S. yaanense* (from 1 to 321 bp).

A non-coding region between *rrnS* and *trnI* was identified as the main non-coding region (mNCR, or AT-rich region) for the two *Sinopotamon* crab species. The mNCR consisted of 1231 bp in *S. yaanense* (with 78.9% A + T content), whereas it consisted of 1194 bp in *S. yangtsekiense* (79.3%). The alignment of the entire sequences of mNCR revealed a high similarity between the two *Sinopotamon* species in two dormains (Suppl. material 1), characterized by a highly conserved central domain and extended termination associated sequences (ETAS).

The overall A + T content of 13 PCGs, calculated from the coding strand of each gene, was 70.7% in *S. yaanense* and 71.8% in *S. yangtsekiense* (Table 2). *Cox1* had the lowest A + T content (64.8%) and *nad4* the highest (74.5%) in *S. yaanense*, whereas for *S. yangtsekiense cox1* the lowest (65.6%) and *nad6* the highest (77.0%). Of the 13 PCGs, nine genes started with the initiation codons ATG (*cox1*, *cox2*, *atp8*, *cox3*, *nad4*, *nad4L*, *nad5*, *cob* and *nad2*); the others began with ATA, ATC and ATT (*atp6*, *nad3*, *nad6* and *nad1*) (Table 2). Meanwhile, ten genes used the typical termination codons TAG and TAA (*cox1*, *atp6*, *atp8*, *cox3*, *nad3*, *nad4*, *nad4L*, *cob*, *nad1* and *nad2*); the remaining genes used incomplete stop codons T (*cox2*, *nad5* and *cob*) (Table 2).

**Table 2.** Nucleptide composition in 13 PCGs of the *Sinopotamon yaanense* and *S. yangtsekiense* mitogenomes.

	Species	Base composition(%)				A+T (%)	Length (nt)	Start codons	Stop codons
		A	T	G	C				
*cox1*	*S. yaanense*	27.60	37.20	15.70	19.50	64.80	1539	ATG	TAA
	*S. yangtsekiense*	27.70	37.90	16.00	18.30	65.60	1539	ATG	TAA
*cox2*	*S. yaanense*	31.88	37.26	11.79	19.07	69.14	688	ATG	T
	*S. yangtsekiense*	31.43	39.62	12.13	16.81	71.05	685	ATG	T
*atp8*	*S. yaanense*	26.14	47.71	7.19	18.95	73.86	159	ATG	TAG
	*S. yangtsekiense*	27.45	49.02	4.58	18.95	76.47	159	ATG	TAG
*atp6*	*S. yaanense*	30.06	40.63	10.42	18.90	70.68	675	ATT	TAA
	*S. yangtsekiense*	29.76	41.52	10.71	18.01	71.28	675	ATT	TAA
*cox3*	*S. yaanense*	26.62	39.67	14.45	19.26	66.29	792	ATG	TAA
	*S. yangtsekiense*	27.25	40.43	13.81	18.50	67.68	792	ATG	TAA
*nad3*	*S. yaanense*	27.64	44.73	11.68	15.95	72.36	354	ATC	TAG
	*S. yangtsekiense*	27.64	44.44	11.11	16.81	72.08	354	ATC	TAG
*nad5*	*S. yaanense*	30.84	41.61	18.75	8.80	72.45	1729	ATG	T
	*S. yangtsekiense*	30.84	41.61	18.75	8.80	72.45	1729	ATG	T
*nad4*	*S. yaanense*	32.35	42.19	17.36	8.10	74.54	1335	ATG	TAG
	*S. yangtsekiense*	30.94	42.92	18.35	7.79	73.86	1338	ATG	TAA
*nad4L*	*S. yaanense*	26.00	46.33	22.00	5.67	72.33	303	ATG	TAA
	*S. yangtsekiense*	27.00	47.33	20.33	5.33	74.33	303	ATG	TAA
*nad6*	*S. yaanense*	26.91	47.39	6.63	19.08	74.30	504	ATT	TAA
	*S. yangtsekiense*	27.38	49.60	6.55	16.47	76.98	507	ATT	TAA
*cob*	*S. yaanense*	27.43	40.39	12.35	19.84	67.81	1135	ATG	T
	*S. yangtsekiense*	27.43	41.53	12.70	18.34	68.96	1135	ATG	T
*nad1*	*S. yaanense*	26.82	45.09	19.23	8.87	71.90	939	ATA	TAA
	*S. yangtsekiense*	27.67	45.09	18.48	8.76	72.76	939	ATA	TAA
*nad2*	*S. yaanense*	28.44	45.41	7.49	18.66	73.85	1005	ATG	TAA
	*S. yangtsekiense*	29.24	46.11	7.58	17.07	75.35	1005	ATG	TAA
*total*	*S. yaanense*	28.36	42.74	13.46	15.43	70.67	11157		
	*S. yangtsekiense*	28.59	43.63	13.16	14.61	71.81	11160		

### Gene rearrangement

**Figure 2.**
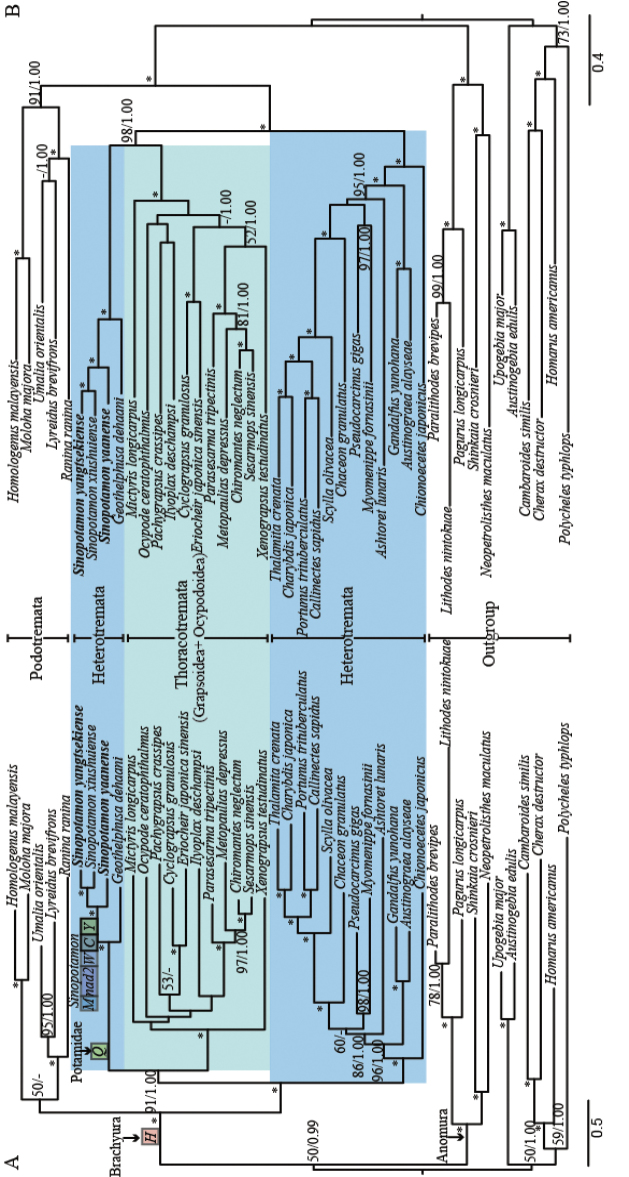
Phylogenetic analyses derived for brachyurans using the maximum likelihood (ML) analyses and Bayesian inferences (BI) using dataset A (13 PCGs) and dataset B (13 PCGs + two rRNAs). Branch lengths and topologies came from ML analysis. Values at the branches represent BP (Bootstrap value)/BPP (Bayesian posterior probability). 100/1.00 is denoted by an asterisk. The horizontal line stands for BP under 50 or BPP under 0.9 ML analyses. The gene rearrangement is denoted by the block on (A): (I) the translocation of *trnH* shared by the Brachyura taxa sampled; (II) the transposition of *trnQ* shared by potamid species; (III) the five-gene block, (*trnM-nad2-trnW-trnC-trnY*), translocation shared by three *Sinopotamon* crabs sampled.

Gene orders of *S. yaanense* and *S. yangtsekiense* mitogenomes were identical between both species (Fig. 1). Compared to the general brachyuran pattern, a novel gene order was found for both *Sinopotamon* crab species with translocations of a gene block and a tRNA gene, where the major rearrangement was due to a five-gene block (*trnM-nad2-trnW-trnC-trnY*) translocated out of *trnI-cox1* gene junction and moved into *trnL1-lrRNA* gene junction (i.e. *trnL1*- ***trnM-nad2-trnW-trnC-trnY***-*lrRNA*). The minor rearrangement was attributed to a transposition of *trnQ* that occurred in mitogenome sequences available for potamid crabs (Fig. 2) that moved from a tRNA gene cluster (*I-Q-M*) to *trnV* and *srRNA* gene junction (i.e. *trnV*-***trnQ***-*srRNA*).

### Phylogenetic inference

The phylogenetic trees were reconstructed based on the two different datasets A (13 PCGs) and B (13 PCGs + two rRNAs). Both ML and Bayesian analyses resulted in congruent tree topologies with the exception of some minor difference within “Grapsoidea + Ocypodoidea”. Branch lengths and topologies came from ML analysis. Bootstrap value (BP) and Bayesian posterior probability (BPP) of nodes are shown like BP/BPP in Fig. 2. The Brachyura and Anomura clades grouped together, recovered as a monophyletic Meiura group, but with strong support in only one Bayesian analysis. The homolid crabs and raninid crabs within the Brachyura formed a monophyletic Podotremata clade with strong support from dataset B analysis (BP = 91; BPP = 1.00), but weakly supported by dataset A analysis (BP =50; BPP < 0.9). All trees place the Podotremata clade as a sister group of the eubrachyuran clade with relatively high nodal support. These results provided evidence for the monophyly of Brachyura and Eubrachyura.

Within the Eubrachyura, strong support was obtained for the non-monophyly of Heterotremata, with potamid crabs nesting outside the heterotreme clade ((((Portunoidea + Calappoidea) + (Xanthoidea + Eriphioidea)) + Bythograeoidea) + Majoidea); instead, four potamid crabs appeared to constitute a close affinity to Thoracotremata, and form a robust clade ((Grapsoidea + Ocypodoidea)+Potamidae). All potamids, *S. yaanense*, *S. yangtsekiense* and *S. xiushuiense* constituted a robust clade with *G. dehaani* being sister to this clade in all analyses, with relatively high nodal support (BP = 100 and 100; BPP = 1.00 and 1.00).

## Discussion

The mitogenomic organizations found in the two *Sinopotamon* species are identical to those of the typical decapods (Qian et al. 2011). The A + T content of all 13 PCGs in the two species were obtained, indicating an AT bias (70.7% in *S. yaanense*, and 71.8% in *S. yangtsekiense*) similar to that reported in most of the mitogenomes available for brachyurans (Bai et al. 2016). This high A + T content may be correlated with the strong A + T bias in codon usage (Song et al. 2016; Tan et al. 2017). The initiation codons of 13 PCGs are usual ATN, but the incomplete stop codon T is assigned for three PCGs (Table 2). These variable start codons and incomplete stop codons (T and/or TA) are frequently found in other brachyurans (Yamauchi et al. 2003; Sun et al. 2005; Shi et al. 2015; Bai et al. 2016), and may be completed by polyadenylation during the mRNA maturation process (Ojala et al. 1981; Qiu et al. 2005).

The largest non-coding region between *rrnS* and *trnI* was predicted to be the putative mNCR in the two *Sinopotamon* crab species ranging from 1194 bp (*S. yangtsekiense*) to 1231 bp (*S. yaanense*), where the position is similar to that found in *Geothelphusa dehaani* and other brachyurans (see Segawa and Aotsuka 2005; Bai et al. 2016; Ma et al. 2016a, 2016b). The comparisons of mNCR of the two *Sinopotamon* species revealed a highly conserved structure, including a central domain and ETAS (Supporting information, Suppl. material 1). The similarity of nucleotide composition between the two species is 77% and 70% in these two domains, respectively. A similar characterized structure has been demonstrated in vertebrates (Saccone et al. 1987; Sbisa et al. 1997; Kierstein et al. 2004). The similar structure can be also identified from the largest intergenic spacer consisting of 1221 bp for *S. xiushuiense* mitogenome, although this intergenic spacer was not regarded as the mNCR by Wang et al. (2016). Furthermore, several repeated motifs were identified at the 3’ end of the central domain for three *Sinopotamon* species, including TA (TC), TA (T), CA (TA) and AT (AA). Similar motifs were found in other brachyurans (Pie et al. 2008; Jondeung et al. 2012; Shi et al. 2015; Ma et al. 2016a, 2016b). These results indicate that mNCR could be used as a genetic marker for studies regarding population structure and phylogeographic patterns of related species (Diniz et al. 2005).

Gene arrangements and primary sequences in mitogenomes embrace useful signals in reconstruction of phylogenetic relationships (Boore et al. 1995, 1998; Boore 1999; Sun et al. 2003, 2005; Tan et al. 2017). The brachyuran crabs are among the most specious groups in decapod crustaceans (Ng et al. 2008; Guinot et al. 2013). In terms of gene rearrangement, our results revealed that brachyuran crabs share a translocation of *trnH*, which moved from the *nad5-nad4* gene junction into a major tRNA gene cluster (*A-R-N-S1-E-F*) between the *nad3* and *nad5* genes; this is common for brachyuran mitogenomes (Fig. 2; Yamauchi et al. 2003; Sun et al. 2005; Shi et al. 2015). A translocation of tRNA gene in mitogenome, even if this is minor rearrangement, provides in several cases convincing phylogenetic evidence of deducing phylogenetic relationships among major groups of arthropods (Boore et al. 1995, 1998). The shared rearrangement of *trnH* in brachyurans agrees with the molecular phylogeny presented here and previous studies, which supports the monophyly of Brachyura (Jamieson et al. 1994, 1995; Scholtz and McLay 2009; Qian et al. 2011; van Bakel et al. 2012; Ji et al. 2014; Tsang et al. 2014; Shi et al. 2015). Compared to the ancestral pattern of mitochondrial gene order of brachyurans, we further found that a translocation of *trnQ* was shared by mitogenomes available for the sampled potamids (Fig. 2). Furthermore, a novel rearrangement involving five genes (*trnM-nad2-trnW-trnC-trnY*) was only found in mitogenomes available for *Sinopotamon* species. Common gene rearrangements that possibly act as synapomorphy for phylogenetic estimation have been identified in decapod crustaceans (Shen et al. 2015; Tan et al. 2015, 2017). Here we found that novel gene order attributes act as useful phylogenetic characters for Potamidae. We propose that *trnQ* rearrangement is most likely a synapomorphy in potamid lineages, and the distinct five gene block rearrangement might be a derived pattern for *Sinopotamon* crabs during the long period of evolution of potamid lineages. More gene rearrangement patterns and their evolutionary implications in potamids may be better understood through the comparison of mitogenomes of a broader taxon sampling.

Striking species diversification has challenged the phylogeny of brachyurans and led to a high number of hypotheses about affinities within the Eubrechyura (reviewed in Guinot et al. 2013; Tsang et al. 2014). All primary freshwater crabs, involving potamids, are recognized as true heterotremes (Guinot et al. 2013). The monophyletic nature of Potamidae is confirmed by morphological and molecular analyses (reviewed in Yeo et al. 2008). However, its phylogenetic placement remains controversial based on morphological characters and molecular phylogeny (Dai 1999; Von Sternberg et al. 1999; Von Sternberg and Cumberlidge 2001). Our results show that these heterotreme crabs, *G. dehaani*, *S. xiushuiense*, *S. yaanense* and *S. yangtsekiense* are in fact associated more closely with thoracotreme crabs than to the remaining heterotremes. Therefore, we propose a phylogenetic framework for the non-monophyly of Heterotremata. However, in the combined gene tree presented by Tsang et al. (2014), the potamid clade along with the other primary freshwater crabs align with heterotreme crabs rather than with thoracotreme crabs (without strong support). Interrelationships of major clades within the Eubrachyura remain one of the most contentious issues in systematics today. Von Sternberg and Cumberlidge (2001) suggested that families of Old World freshwater crabs including Potamonautidae endemic to the Afrotropical region, the Potamidae and Gecarcinucidae endemic to Oriental region, should be placed in Thoracotremata and aligned with gecarcinids, grapsoids and ocypodoids s.l. based on inferences of multiple adult morphological characters. Recent molecular phylogenetic analyses based on DNA also suggested that the Heterotremata is non-monophyletic (Schubart et al. 2000; Porter et al. 2005; Ahyong et al. 2007; Mahon and Neigel 2008; Bracken et al. 2009; Ji et al. 2014; Xing et al. 2016). In this context, rapid and efficient methods using next-generation sequencing could recover sufficient mitogenomes through far greater taxon sampling and build a high-resolution phylogenetic relationship within the Brachyura.

## Supplementary materials

Supplementary material 1 Figure S1 Data type: TIF image file Explanation note: Comparisons of the consensus sequence and variable sites in the entire mNCR for *Sinopotamon yaanense*, *S. yangtsekiense* and *S. xiushuiense*. The conserved central domain is grey shaded, and the extended termination associated sequences (ETAS) is underlined. This dataset is made available under the Open Database License (http://opendatacommons.org/licenses/odbl/1.0/). The Open Database License (ODbL) is a license agreement intended to allow users to freely share, modify, and use this Dataset while maintaining this same freedom for others, provided that the original source and author(s) are credited.Xing Yuhui, Zhou Lijun, Hou Yue, Wang Xiaoqi, Zhang Chen, Zhang Huilun, Wang Ruoran, Pan Da, Sun Hongying
