# Supplementary figures and images for: Complete mitochondrial genomes from two species of Chinese freshwater crabs of the genus Sinopotamon recovered using next-generation sequencing reveal a novel gene order (Brachyura, Potamidae)
Source: Zookeys. 2017 Oct 2;(705):41–60. doi: 10.3897/zookeys.705.11852 (PMC5674035; doi:10.3897/zookeys.705.11852)

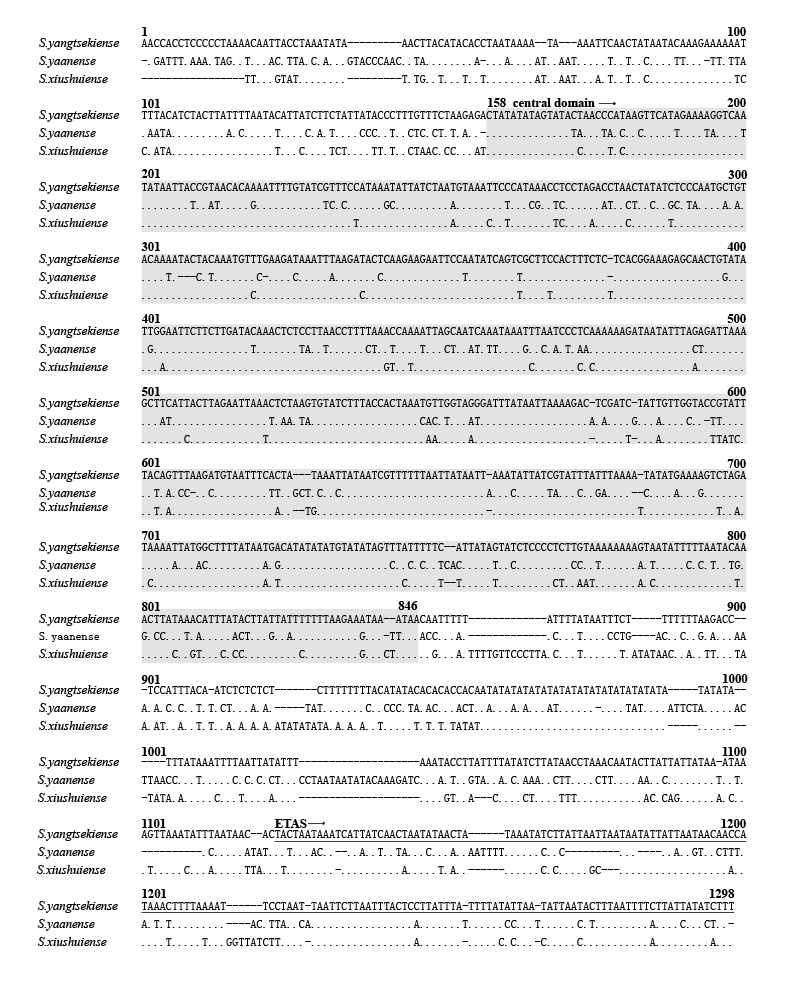

Supplement: Supplementary material 1 — Figure S1 [file zookeys-705-041-s001.tif]
